# Supplementary material for: Using causal machine learning and real world data to improve dose response decision making for secukinumab in psoriatic arthritis
Source: Sci Rep. 2026 Apr 11;16:12186. doi: 10.1038/s41598-026-47976-8 (PMC13076753; doi:10.1038/s41598-026-47976-8)
Supplement: Supplementary file 1 — Supplementary Material 1 [file 41598_2026_47976_MOESM1_ESM.docx]

**SUPPLEMENT**

**METHODS**

**Variables Construction**

For this analysis MOS data was aggregated into a single variable by averaging the raw item responses of 12 items from the MOS Sleep Scale. For 10 items, higher values indicate better sleep quality. One item indicates better sleep with lower values but was not reversed for calculation. Another item, which measures average hours of sleep per night, is interpreted in a u-shaped manner, if 8 hours is considered optimal. Although this approach is not perfectly accurate, it provides a straightforward and efficient way to assess overall sleep quality.

Additionally, a variable for Patient Global Assessment (PGA) was derived by averaging the responses on the three VAS dimensions: Global Disease Activity, Global Pain and Nightly Pain.

Weighted Joint Count (WJC) was calculated as a weighted average of Tender Joint Count 68 (TJC68) and Swollen Joint Count 66 (SJC66) as follows: WJC = ⅔ * TJC68 + ⅓ * SJC66. The weights were derived from the DAS28 (CRP) formula DAS28 (CRP) = 0.56*√(TJC28) + 0.28*√(SJC28) + 0.014*GH + 0.36*ln(CRP+1) + 0.96 [Wells et al. 2009] in which the coefficient for TJC28 = 0.56 is twice the coefficient for SJC28 = 0.28.

**Causal analysis**

Using causal analysis, researchers can evaluate treatment effect heterogeneity which, in turn, enhances their comprehension of the factors influencing treatment outcomes. Additionally, it may facilitate the identification of subgroups that exhibit better response to the treatment [Kent et al 2020].

Estimating the treatment effect using observational data provides an alternative method for replicating randomized control trials (RCTs) and determining causation. However, observational studies do not have the advantage of random assignment, which makes it crucial to navigate the complexities of confounding variables and selection bias to make inferences about causation. Causal analysis can help address these challenges by providing a structured framework for untangling causal relationships from observed correlations [Ling Y et al 2023]. In most scenarios, a study population demonstrates heterogeneity, which implies that there are variations in individual characteristics that could potentially influence the effects of a treatment. In simple terms, the impact of a treatment can vary significantly within specific subgroups, deviating from the overall average treatment effect. These variations in the direction and magnitude of treatments for individuals can be explained by causal mechanisms [Yao Y, et al. 2021].

One important concept for causal analysis is unconfoundedness (that we have tracked all relevant confounders). A confounding variable (or confounder), is a factor that affects both the treatment and the outcome, leading to a misleading association. Many causal algorithms (such as the ‘Double Machine Learning’ models used in our study) are under the assumption of unconfoundedness. Although it is often impossible to measure all confounders in practice, they are not a problem if we know and track them. The selection of confounders in this study was primarily based on clinical expertise, but various combinations of covariates were also experimented with as potential confounders to observe their impact on the results.

The last important fact is that causal inference is an unsupervised learning technique that makes the task of validation challenging. The objective of causal inference is to comprehend the results of different courses of action. It is only when confirming the resilience of an estimate in consideration of unverified assumptions that the causal estimates can be deemed valid and unbiased. Typically, the robustness of the model is assessed using statistical methods, followed by validation through the insights of subject matter experts.

**Model Selection**

**Performance metrics:** The first stage models were built based on the observed outcomes, allowing, in principle, for the measurement of performance. However, the CausalAnalysis class did not directly offer this possibility nor provide a means to influence the model-fitting process. **We therefore applied a different strategy for model optimization, which is described below.**

**Machine learning optimization and validation pipeline:** To fine-tune the model using the CausalAnalysis class, we adjusted several parameters to optimize performance. The parameters and their roles are as follows:

*heterogeneity_model*: This parameter determines the type of model used for the final heterogeneous treatment effect (stage 2). We considered two options: 'linear' and 'forest'. The 'linear' model estimates the treatment effect as a linear function of the heterogeneity features, while the 'forest' model uses a forest-based approach to compute the effect from these features. Although linear models demonstrated marginally superior performance on the metric described below, we ultimately chose the 'forest' model, as a non-linear relationship between covariates and treatment seemed to be more plausible. *feature_inds*: The feature selection for the ML model was determined through a combination of preliminary SHAP analyses, literature research, and model tuning. Initially, SHAP analysis was used to identify the most influential features, which were then cross-referenced with findings from relevant studies to ensure their significance. Finally, model tuning was performed to refine the feature set, ensuring optimal performance and relevance to the clinical context. *heterogeneity_inds*: This parameter specifies the subset of features used as control variables (X), with the remaining features treated as confounders (W). This distinction helps in accurately estimating the treatment effect by controlling for confounding variables. *cv*: This stands for cross-validation, and we used either 5 or 10 folds (k-fold cross-validation) to ensure the robustness of our model. Cross-validation helps in assessing the model's performance and generalizability. *mc_iters*: This parameter represents the number of times to rerun the first stage models, ranging from 3 to 20 iterations. By rerunning the first stage models with new splits each time, we aim to reduce the variance of the causal model nuisances, making the nuisance estimates from the first-stage models less noisy.

To guide the tuning process, we trained 10 models using the same set of 10 random numbers and averaged the p-values of the treatment variable, with a smaller average p-value being preferable. We selected the final model based on this indicator. This approach helps in mitigating the risk of overfitting by providing a more stable estimate of the model's performance.

**RESULTS**

**Supplementary Figure 1: Observed PsAID distribution on baseline and PsAID change after 16-28 weeks**

**Supplementary Figure 2: Observed PsAID treatment effect by dose as a function of status of smoking**
